# C2 Slope as an Independent Predictor of Cervical Lordosis Loss Following Laminoplasty

**DOI:** 10.1111/os.70264

**Published:** 2026-01-30

**Authors:** Bin Zheng, Panfeng Yu, Zhenqi Zhu, Yan Liang, Haiying Liu

**Affiliations:** ^1^ Spine Surgery Peking University People's Hospital Beijing China

**Keywords:** C2 slope, cervical lordosis, laminoplasty, predictive factor, sagittal alignment

## Abstract

**Objective:**

Postoperative loss of cervical lordosis remains a common and clinically relevant complication following laminoplasty, negatively affecting neck pain, neurological recovery, and long‐term sagittal balance. However, reliable and easily applicable preoperative predictors for identifying patients at high risk of cervical lordosis deterioration remain limited. This study aims to investigate whether preoperative C2 slope (C2S) independently predicts cervical lordosis deterioration following laminoplasty.

**Methods:**

This retrospective cohort study included 179 patients who underwent cervical laminoplasty for cervical spondylotic myelopathy at our institution between April 2014 and December 2020, with a minimum follow‐up of 24 months. Radiological parameters including C2‐7 Cobb angle, cervical sagittal vertical axis (cSVA), C7 slope, and C2S are measured preoperatively and at final follow‐up. Patients are divided into lordosis deterioration group (> 5°) and control group (≤ 5°). B Between‐group comparisons are performed using independent‐samples *t* tests and *χ* ^2^ tests. Binary logistic regression analysis is conducted to identify independent predictors of postoperative cervical lordosis loss. Receiver operating characteristic (ROC) curve analysis is used to evaluate predictive performance and determine the optimal cutoff value.

**Results:**

The lordosis loss group (*n* = 55) shows significantly higher preoperative C2S (14.57° ± 3.47° vs. 9.52° ± 7.30°, *p* < 0.001), lower preoperative Cobb angle (13.01° ± 4.91° vs. 16.1° ± 6.50°, *p* < 0.001), and greater cSVA (2.58 ± 1.45 cm vs. 2.13 ± 1.42 cm, *p* = 0.027) compared to controls. The lordosis loss group demonstrates worse postoperative neck pain VAS scores (3.31 ± 1.63 vs. 2.40 ± 1.56, *p* < 0.001) and slightly lower JOA scores (15.45 ± 1.14 vs. 15.78 ± 1.12, *p* = 0.037). Multivariate analysis reveals preoperative C2S as the only independent predictor (OR = 1.176, *p* < 0.001), with 11.49 as cutoff value for C2S.

**Conclusion:**

Elevated preoperative C2S independently predicts postoperative cervical lordosis deterioration. C2S measurement provides a simple, effective tool for identifying high‐risk patients and optimizing surgical planning.

## Graphical abstract

Cervical radiographic measurements demonstrate that the C2 slope is an independent predictor of cervical lordosis loss after laminoplasty, aiding preoperative risk assessment and surgical decision‐making.

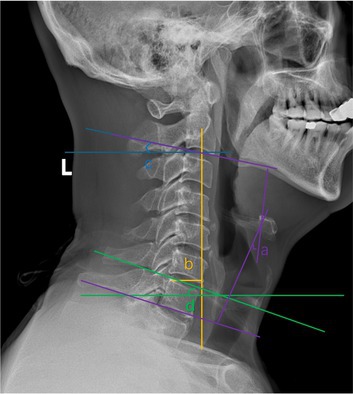

## 1 Introduction

Cervical lordosis plays a crucial role in maintaining head posture, ensuring spinal cord relaxation, and balancing overall body posture. If normal cervical lordosis is lost postoperatively, resulting in cervical kyphosis or even sagittal imbalance, patients often experience exacerbated neck and shoulder pain, poor neurological recovery, and compromised quality of daily life [1, 2, 3]. Therefore, preoperative identification of patients at higher risk for postoperative cervical lordosis loss has significant clinical implications for surgical strategy development and rehabilitation planning [4, 5, 6].

Previous studies propose several potential influencing factors. For instance, lower preoperative cervical Cobb angle may increase the risk of postoperative kyphosis, with some studies reporting that patients with preoperative C2‐7 Cobb angle less than approximately 7° are more prone to developing postoperative cervical kyphosis [7]. Additionally, morphological parameters of the cervicothoracic junction, such as T1 slope (T1S), are associated with postoperative cervical lordosis changes [8]. However, relying solely on these indicators has limitations. For example, Cobb angle only reflects the cervical spine's intrinsic curvature without considering head and thoracic posture; while T1S is important, it requires combination with cervical lordosis (i.e., T1S‐CL mismatch) for comprehensive cervical balance assessment, making it operationally complex [9, 10].

In recent years, C2 slope (C2S) emerges as a novel cervical sagittal alignment parameter. C2S is defined as the angle between the C2 inferior endplate and the horizontal plane [11, 12, 13]. From mathematic perspective, C2S essentially approximates “T1S‐CL” [14]. Therefore, C2S can be viewed as a single integrated indicator of upper and lower cervical alignment relationships, reflecting the matching degree between cervical lordosis and thoracic inclination. Some scholars propose that elevated C2S indicates insufficient cervical lordosis to match greater thoracic inclination, requiring excessive atlantoaxial hyperextension to maintain horizontal gaze, thus suggesting potential cervical sagittal imbalance [13].

Despite its theoretical advantages, the clinical significance of preoperative C2 slope in predicting postoperative cervical lordosis deterioration following laminoplasty remains insufficiently investigated. Therefore, the objectives of this study are as follows:

(i) to evaluate the relationship between preoperative C2 slope and postoperative cervical lordosis loss after laminoplasty; (ii) to determine whether C2 slope independently predicts postoperative cervical lordosis deterioration after adjustment for conventional sagittal alignment parameters; and (iii) to identify an optimal C2 slope cutoff value for preoperative risk stratification.

By clarifying the predictive value of C2 slope, this study aims to provide a simple and clinically practical radiological marker to facilitate preoperative assessment, individualized surgical planning, and future development of alignment‐preserving strategies in patients undergoing cervical laminoplasty.

## 2 Methods

### 2.1 Study Design

This is a retrospective cohort study. Inclusion criteria comprise patients who undergo laminoplasty for cervical degenerative diseases at our institution from April 2014 to December 2020, with complete follow‐up of at least 24 months. Patients with previous cervical surgery history, incomplete imaging data, or poor image quality are excluded. A total of 179 patients meet the criteria and are included in the analysis. This study is approved by Peking University People's Hospital ethical committee (2024PHB156).

### 2.2 Surgery Details

All patients undergo standard open‐door cervical laminoplasty performed by the same surgeon. After induction of general anesthesia and prone positioning with the head fixed in a neutral posture, a posterior midline incision is made. The paraspinal muscles are dissected subperiosteally to expose the laminae and lateral masses at the planned decompression levels. The decompression range is determined according to preoperative MRI findings and the extent of spinal cord compression. The lamina on the open side is completely cut using a high‐speed burr, while the hinge side is thinned to preserve cortical continuity. The lamina is then elevated to form an “open‐door” configuration and fixed to the lateral mass with titanium miniplates. After confirming adequate spinal cord decompression and achieving meticulous hemostasis, a suction drain is placed, and the wound is closed in layers. Postoperatively, patients wear a soft cervical collar for 2–4 weeks, and gentle neck and shoulder exercises are initiated after drain removal.

### 2.3 Data Collection

**FIGURE 1:**
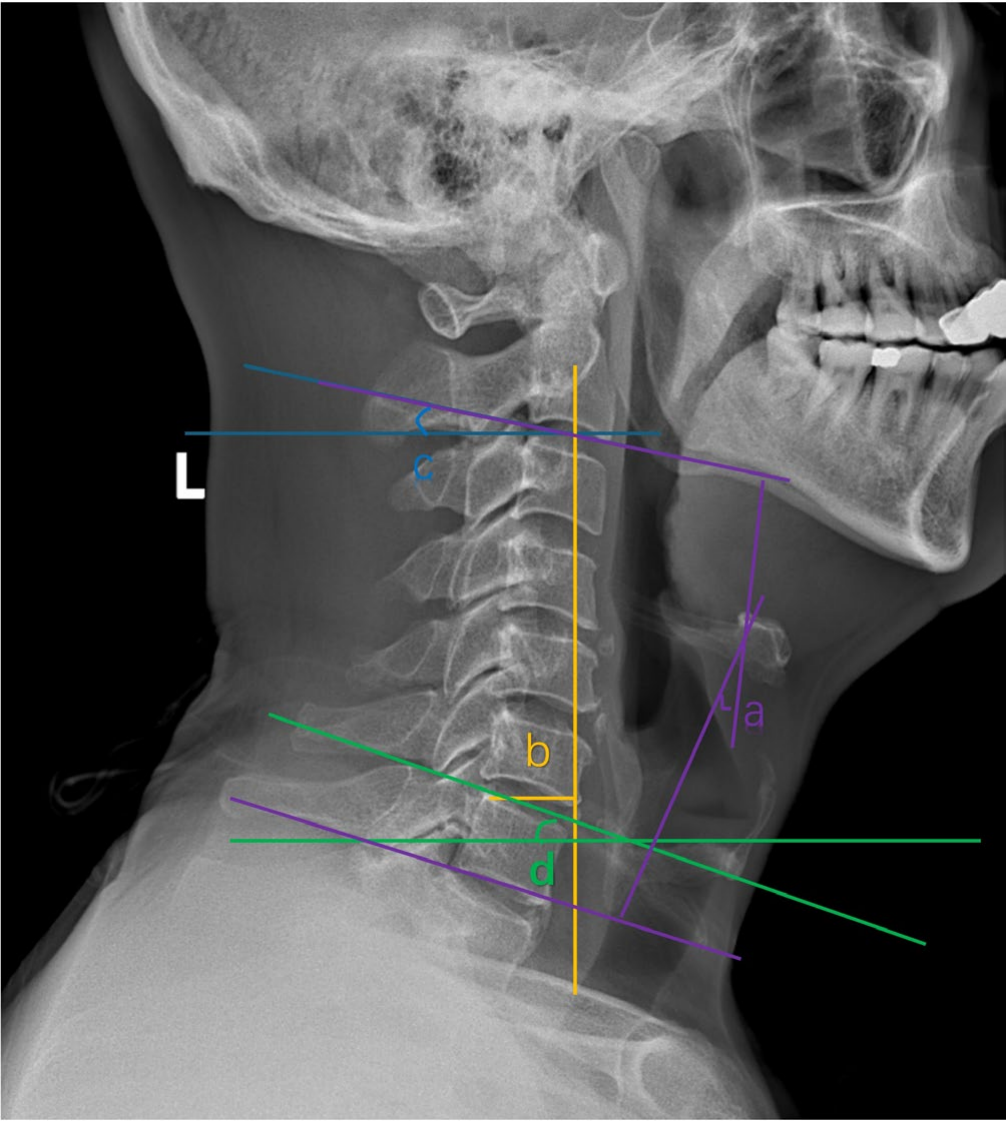
Measurement of cervical sagittal parameters on lateral radiograph. Illustration of radiological parameter definitions. (a) C2–7 Cobb angle: The angle between the inferior endplates of C2 and C7, representing overall cervical lordosis. (b) cSVA: The horizontal distance between the C2 plumb line and the posterior‐superior corner of the C7 vertebral body; (c) C2 slope (C2S): The angle between the C2 inferior endplate and the horizontal line; (d) C7 slope (C7S): The angle between the C7 superior endplate and the horizontal line.

General patient data (gender, age, follow‐up duration) are collected. All patients undergo standing lateral cervical spine X‐ray examination preoperatively and during postoperative follow‐up. The following radiological parameters are measured(shown in Figure 1): cervical C2‐7 Cobb angle (angle between C2 inferior endplate and C7 inferior endplate), cervical sagittal vertical axis distance (cSVA, horizontal distance between C2 plumb line and C7 posterior‐superior endplate vertical line, cm), C7 slope (C7S, angle between C7 superior endplate and horizontal plane, °), C2 slope (C2S, angle between C2 inferior endplate and horizontal plane, °), and cervical range of motion (ROM, difference between maximum extension and flexion C2‐7 Cobb angles, °). Clinical indicators include Japanese Orthopaedic Association (JOA) cervical myelopathy score and neck pain Visual Analog Scale (VAS), both assessed preoperatively and at final follow‐up.

Postoperative loss of cervical lordosis (LCL) is defined as a decrease in the C2–7 Cobb angle of more than 5° at the final follow‐up compared with the preoperative measurement. This threshold is selected according to previous studies, which commonly adopt a > 5° loss as a clinically meaningful deterioration in cervical alignment [15, 16]. Patients are divided into two groups: postoperative cervical lordosis loss > 5° group (deterioration group) and postoperative lordosis loss ≤ 5° group (control group). Informed consent is obtained from all patients, and the study protocol is approved by the hospital ethics committee.

Inter‐ and intra‐observer reliability of radiological measurements was assessed using intraclass correlation coefficients (ICCs). Two independent spine surgeons, blinded to clinical outcomes, performed all measurements, and one observer repeated the measurements after a 2‐week interval. ICCs were calculated using a two‐way random‐effects model with absolute agreement. The overall ICC demonstrated excellent reliability, with an inter‐observer ICC of 0.90 and an intra‐observer ICC of 0.93 for all radiological parameters.

### 2.4 Statistical Analysis

Data analysis is performed using SPSS 26.0 statistical software. Quantitative data are expressed as mean ± standard deviation, with between‐group comparisons using independent samples *t*‐test; categorical data are expressed as numbers and percentages, with between‐group comparisons using *χ* ^2^ test or Fisher's exact test. First, differences in preoperative baseline characteristics, radiological parameters, and postoperative clinical outcomes are compared between the lordosis loss group and control group (see Table 1). Subsequently, binary logistic regression analysis is used to identify independently associated preoperative factors, with cervical lordosis loss > 5° as the dependent variable. Variables included in the regression are preoperative parameters with *p* < 0.1 in univariate analysis (such as Cobb angle, cSVA, C2S) and potential confounders. Logistic regression employs stepwise variable selection, with variables finally entering the equation after collinearity adjustment and their Odds Ratios (OR) with 95% confidence intervals expressed as exponentials of regression coefficients. Finally, for significant predictors identified, receiver operating characteristic (ROC) curves are plotted and area under the curve (AUC) calculated to evaluate discrimination efficacy for lordosis loss. The optimal cutoff value at maximum Youden index is determined, with corresponding sensitivity and specificity calculated. Significance level is set at *α* = 0.05.

**TABLE 1:** Baseline characteristics and postoperative outcomes stratified by postoperative Cobb angle deterioration.

Parameters	Postoperative Cobb < 5* group (*n* = 124)	Postoperative Cobb > 5* group (*n* = 55)	*p*
Gender (male/female)	58/66	28/27	0.63
Follow‐up time (months)	26.84 ± 1.96	27.07 ± 1.71	0.091
Smoke (no/yes)	109/15	46/9	0.48
Diabetes (no/yes)	106/18	45/10	0.52
*Preoperative parameters*			
Preoperative Cobb angle (°)	16.1 ± 6.50	13.01 ± 4.91	< 0.001
Preoperative cSVA (cm)	2.13 ± 1.42	2.58 ± 1.45	0.027
Preoperative C7S (°)	23.74 ± 4.29	23.59 ± 4.11	0.412
Preoperative C2S (°)	9.52 ± 7.30	14.57 ± 3.47	< 0.001
Preoperative ROM (°)	22.77 ± 4.68	22.27 ± 4.56	0.254
Preoperative JOA	8.90 ± 1.97	9.13 ± 1.98	0.234
Preoperative VAS	5.5 ± 1.80	5.58 ± 1.77	0.39
*Postoperative parameters*			
Postoperative Cobb angle (°)	12.26 ± 7.09	6.71 ± 4.90	< 0.001
Postoperative cSVA (cm)	2.80 ± 1.60	3.35 ± 1.55	0.018
Postoperative C7S (°)	25.87 ± 4.46	25.35 + 4.34	0.233
Postoperative C2S (°)	15.40 ± 8.73	19.15 ± 5.96	< 0.001
Postoperative ROM (°)	19.79 ± 5.19	19.33 ± 4.88	0.29
Postoperative JOA	15.78 ± 1.12	15.45 ± 1.14	0.037
Postoperative VAS	2.4 ± 1.56	3.31 ± 1.63	< 0.001

## 3 Results

### 3.1 Baseline Characteristics and Preoperative Parameters

Comparison results of general characteristics, preoperative radiological parameters, and postoperative follow‐up outcomes between the two groups are shown in Table 1. No statistically significant differences are found between the lordosis loss group and control group in gender composition, mean follow‐up duration, smoking, diabetes, and other general data (*p* > 0.05). Among preoperative radiological parameters, the lordosis loss group has a mean preoperative C2‐7 Cobb angle of 13.01° ± 4.91°, significantly smaller than the control group's 16.1° ± 6.50° (*p* < 0.001), suggesting that smaller preoperative cervical lordosis may be a risk factor for postoperative lordosis loss. Additionally, the lordosis loss group shows more pronounced preoperative cervical sagittal vertical axis anterior displacement, with preoperative cSVA of 2.58 ± 1.45 cm, significantly greater than the control group's 2.13 ± 1.42 cm (*p* = 0.027). Mean preoperative C7 slope (approximately reflecting T1 slope) is similar between groups (23.59° vs. 23.74°), with no statistically significant difference (*p* = 0.412). Notably, preoperative C2 slope differs significantly between groups: the lordosis loss group has a preoperative C2S as high as 14.57° ± 3.47°, significantly greater than the control group's 9.52° ± 7.30° (*p* < 0.001). This indicates that patients with higher preoperative C2S are more prone to postoperative cervical lordosis loss. No significant differences are found between groups in preoperative cervical ROM or JOA scores (*p* > 0.05). Additionally, preoperative neck pain VAS scores show no significant difference in Table 1.

### 3.2 Postoperative Radiological and Clinical Outcomes

Regarding postoperative final follow‐up radiological and clinical indicators, the lordosis loss group shows a marked decrease in cervical lordosis compared to preoperative values, with a final C2‐7 Cobb angle of only 6.71° ± 4.90°, significantly smaller than the control group's 12.26° ± 7.09° (*p* < 0.001). The lordosis loss group's reduced cervical lordosis is accompanied by cervical anterior displacement, exacerbating sagittal imbalance, with a final cSVA increasing to 3.35 ± 1.55 cm, still significantly greater than the control group's 2.80 ± 1.60 cm (*p* = 0.018). No significant difference is found in the final C7 slope between groups (approximately 25°, *p* = 0.233). The lordosis loss group maintains a high final C2S (19.15° ± 5.96°), significantly higher than the control group's 15.40° ± 8.73° (*p* < 0.001), indicating more pronounced postoperative upper cervical compensatory hyperextension in the lordosis loss group. Furthermore, regarding clinical outcomes, the lordosis loss group has a final follow‐up JOA score of 15.45 ± 1.14, slightly lower than the control group's 15.78 ± 1.12 (*p* = 0.037), indicating slightly poorer neurological functional improvement. Neck pain VAS score in the lordosis loss group is 3.31 ± 1.63, significantly higher than the control group's 2.40 ± 1.56 (*p* < 0.001), suggesting that cervical lordosis loss may lead to more pronounced neck pain symptoms.

### 3.3 Multivariate Logistic Regression Analysis

Multivariate logistic regression analysis results are shown in Table 2. Preoperative variables included in the analysis are C2‐7 Cobb angle, cSVA, and C2S. After adjusting for mutual influences among factors, results show that preoperative C2S is the only independent predictor of postoperative cervical lordosis deterioration. In contrast, while preoperative Cobb angle and cSVA are associated with outcomes in univariate analysis, they show no significant independent predictive role in multivariate regression (preoperative Cobb angle OR = 0.992, *p* = 0.836; preoperative cSVA OR = 1.257, *p* = 0.063). This demonstrates that when considering multiple factors simultaneously, only C2S has independent predictive value for postoperative cervical deterioration.

**TABLE 2:** Logistic regression analysis of preoperative factors associated with postoperative Cobb angle > 5.

	*B*	SE	Wald	df	Sig.	Exp(*B*)
Preoperative C2S	0.162	0.044	13.806	1	< 0.001	1.176
Preoperative Cobb	−0.008	0.037	0.043	1	0.836	0.992
Preoperative cSVA	0.228	0.123	3.462	1	0.063	1.257
Constant	−3.271	1.000	10.691	1	0.001	0.038

### 3.4 ROC Curve Analysis and Predictive Performance

**FIGURE 2:**
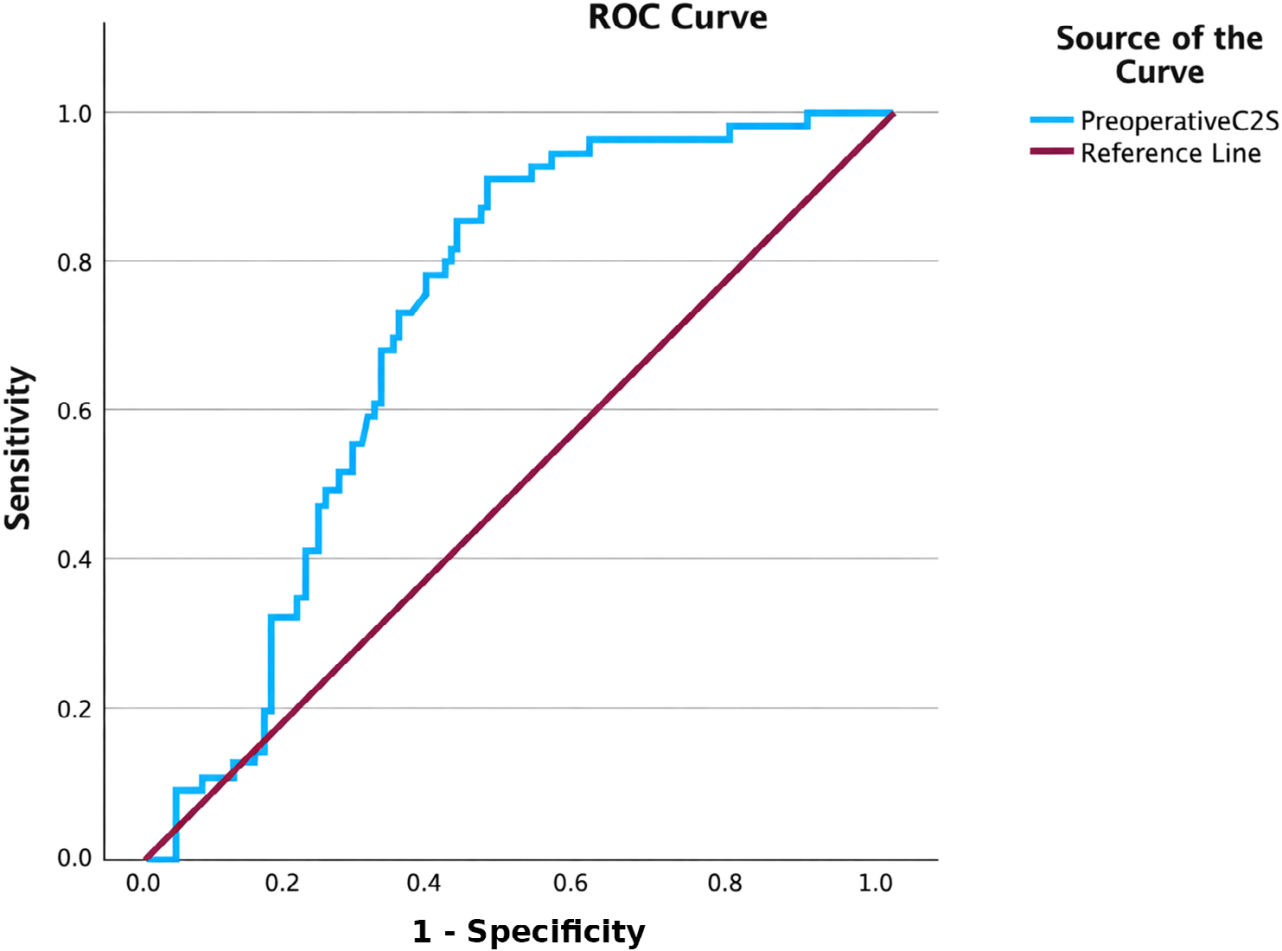
Receiver operating characteristic (ROC) curve of preoperative C2 slope (C2S) for predicting postoperative cervical lordosis loss (> 5°). The model shows AUC = 0.726 (95% CI 0.641–0.811; *p* < 0.001), indicating good discrimination. The optimal cutoff is C2S = 11.49°.

To further evaluate the predictive value of C2S for postoperative cervical lordosis deterioration, an ROC curve is plotted. As shown in Figure 2, the area under the curve for preoperative C2S is 0.726 (95% CI 0.641–0.811; *p* < 0.001). The optimal C2S cutoff value determined by the Youden index method is 11.49°. At this threshold, the sensitivity for predicting postoperative Cobb deterioration is 92.1%, and the specificity is approximately 55.6%.

## 4 Discussion

In this retrospective cohort study, we demonstrate that preoperative C2 slope is an independent predictor of postoperative cervical lordosis deterioration following laminoplasty. Patients with elevated preoperative C2S exhibit significantly greater postoperative loss of cervical lordosis, increased sagittal imbalance, worse neck pain, and slightly inferior neurological recovery. Multivariate analysis confirms that C2S remains the only independent radiological predictor after adjustment for conventional sagittal parameters, and ROC analysis identifies a cutoff value of 11.49° for preoperative risk stratification. These findings highlight the clinical value of C2 slope as a simple and effective marker for identifying patients at high risk of postoperative cervical alignment deterioration.

### 4.1 Association Between Preoperative C2 Slope and Postoperative Cervical Lordosis Loss

This study confirms through retrospective analysis that elevated preoperative C2 slope (C2S) is an independent predictor of postoperative cervical lordosis deterioration. Compared to cervical anatomical parameters (such as Cobb angle) or single balance indicators (such as cSVA), C2S shows a stronger correlation in predicting postoperative cervical sagittal instability. This finding aligns with cervical biomechanical characteristics: C2S, as an upper cervical postural parameter, reflects the degree of head hyperextension required to maintain horizontal gaze. When preoperative C2S is significantly elevated, it indicates that the patient's upper cervical spine is hyperextended to compensate for insufficient lower cervical lordosis or excessive thoracic inclination. This compensatory state may make physiological cervical lordosis maintenance more dependent on muscle balance and ligament tension [17, 18, 19]. Postoperatively, especially after laminoplasty, changes in posterior cervical muscle‐bone stability structures make cervical lordosis more prone to collapse or loss [20, 21, 22]. In this study, we observe that patients with high preoperative C2S show more pronounced postoperative cervical lordosis loss, accompanied by further upper cervical hyperextension (postoperative C2S remains high) and exacerbated cervical anterior displacement (increased cSVA), supporting the proposed mechanism.

### 4.2 C2 Slope as an Independent Predictor Compared With Conventional Sagittal Parameters

Compared to other radiological indicators, C2S's advantage as a predictor lies in its comprehensiveness and simplicity. First, C2S integrates information about both cervical lordosis angle and thoracic inclination angle. The previously commonly used “T1 slope minus cervical lordosis (T1S‐CL)” can quantify the mismatch between cervical and thoracic alignment but requires measuring two parameters with high image quality requirements. C2S requires measuring only the single indicator of C2 inclination angle, making it operationally simpler while being highly correlated with T1S‐CL numerically, serving as a surrogate indicator [18, 23]. Second, compared to simple Cobb angle or cSVA, C2S more comprehensively reflects overall cervical‐trunk balance: patients with large Cobb angles may still have imbalance risk if T1S is simultaneously large; normal cSVA may mask cervical instability through cranial compensation. Elevated C2S often indicates potential mismatch; even with temporarily normal Cobb angle and cSVA, it suggests possibility of future decompensation. The logistic regression results in this study also show that only C2S is independently significant in the multivariate model, further highlighting C2S's predictive advantage over traditional indicators.

### 4.3 Clinical Implications and Cutoff Value of Preoperative C2 Slope

Our study results are consistent with some existing literature reports while providing new clinical insights. Kim et al. find that preoperative C2S is associated with postoperative clinical outcomes in patients undergoing multilevel cervical fusion surgery, proposing that higher C2S might lead to easier cervical muscle group fatigue due to postural imbalance, thereby affecting postoperative quality of life [13]. Besides, Chai reports C2S can better analyze the sagittal alignment of DCK patients than TS‐CL and has good practicability in clinical application and HRQOL evaluation [11]. Our study focuses on laminoplasty, confirming C2S's predictive role for radiological lordosis loss, and further observing that these patients experience concurrent neck pain exacerbation and suboptimal functional improvement with lordosis loss. This suggests that regardless of fusion or nonfusion surgery, patients with preoperative cervical‐trunk mismatch (indicated by high C2S) may face greater risk of postoperative sagittal instability and functional compromise.

Based on our findings, we recommend routine measurement of C2S in preoperative assessment for laminoplasty. For patients with significantly elevated preoperative C2S, heightened vigilance is warranted regarding possible postoperative cervical lordosis loss. For patients with high C2S undergoing laminoplasty, measures should be taken to maintain physiological cervical lordosis; intraoperatively, attention should be paid to preserving posterior cervical muscle group attachments to maintain stability. During postoperative rehabilitation, these high‐risk patients should strengthen cervical muscle strength and postural training, with regular imaging follow‐up to detect and intervene early in progressive cervical lordosis loss. In summary, C2S shows promise as a simple and effective tool to help clinicians optimize perioperative decision‐making and improve patient outcomes.

### 4.4 Strengths

This study benefits from a relatively large laminoplasty cohort with sufficient follow‐up, enabling robust evaluation of postoperative cervical lordosis changes. By focusing on C2 slope as a single, easily measurable parameter, we demonstrate its independent predictive value and practical superiority over composite sagittal indices through multivariate and ROC analyses. The identification of a clinically applicable C2S cutoff further enhances the translational value of our findings for preoperative risk stratification and surgical decision‐making.

### 4.5 Limitations and Future Directions

This study has certain limitations. First, as a retrospective single‐center study, selection bias and uncontrollable confounding factors may exist, limiting causal inference. Second, we primarily focus on radiological parameters without quantitative assessment of soft tissue muscle factors. Third, with follow‐up of approximately 2 years, longer‐term lordosis change trends remain unclear. Future prospective, large‐sample, multicenter studies are needed to validate the generalizability of our results and further explore the effects of C2S‐stratified interventions. For example, whether different surgical approaches or adjunctive measures (such as fusion fixation or postoperative brace protection) can reduce postoperative lordosis loss and improve symptoms in patients with preoperative C2S above specific thresholds will be an important direction for future research.

## 5 Conclusion

Elevated preoperative C2 slope represents a powerful predictor of postoperative cervical lordosis loss following laminoplasty. The integration of C2S measurement into routine preoperative assessment protocols enables early identification of patients at heightened risk for cervical sagittal imbalance, facilitating the development of individualized surgical strategies aimed at preserving physiological cervical curvature and optimizing clinical outcomes.

## Author Contributions

**Bin Zheng** and **Panfeng Yu:** contributed equally to this work. Conception and design: **Bin Zheng**. Acquisition of data: **Panfeng Yu**. Analysis and interpretation of data: **Zhenqi Zhu** and **Yan Liang**. Drafting the article: **Bin Zheng, Panfeng Yu** and **Haiying Liu**. Critically revising the article: **Panfeng Yu** and **Haiying Liu**. All authors have read and agreed to the published version of the manuscript.

## Funding

This study is funded by Peking University People's Hospital (2023HQ05) and Peking University People's Hospital (ZBYS‐ ZYC‐2025‐10), Horizontal Project of Peking University People's Hospital (grant number 2022‐Z‐09), Major Health Special Project of the Ministry of Finance of China (grant number 2127000432), Major Health Special Project of the Ministry of Finance of China (grant number 2127000349), Major Health Special Project of the Ministry of Finance of China (grant number 2127000235), Beijing Municipal Natural Science Foundation (7232191), and Major Health Special Project of the Ministry of Finance of China (grant number 2127000033).

## Ethics Statement

This study was conducted in accordance with the ethical principles of the Declaration of Helsinki. The study was reviewed and approved by the ethics committee of Peking University People's Hospital (2024PHB156).

## Consent

Consent to participate declaration is obtained from patients.

## Conflicts of Interest

The authors declare no conflicts of interest.

## Data Availability

The data that support the findings of this study are available from the corresponding author upon reasonable request.
